# Hypoxia-Induced Non-Coding RNAs Controlling Cell Viability in Cancer

**DOI:** 10.3390/ijms22041857

**Published:** 2021-02-12

**Authors:** Maria Magdalena Barreca, Chiara Zichittella, Riccardo Alessandro, Alice Conigliaro

**Affiliations:** 1 Department of Biomedicine, Neuroscience and Advanced Diagnostics (Bi.N.D.), Section of Biology and Genetics, University of Palermo, 90133 Palermo, Italy; mariamagdalena.barreca@unipa.it (M.M.B.); chiara.zichittella@unipa.it (C.Z.); riccardo.alessandro@unipa.it (R.A.); 2 Institute for Biomedical Research and Innovation (IRIB), National Research Council (CNR), 90146 Palermo, Italy

**Keywords:** hypoxia, HIF, lncRNAs, miRNAs, cancer, proliferation, cell cycle

## Abstract

Hypoxia, a characteristic of the tumour microenvironment, plays a crucial role in cancer progression and therapeutic response. The hypoxia-inducible factors (HIF-1α, HIF-2α, and HIF-3α), are the master regulators in response to low oxygen partial pressure, modulating hypoxic gene expression and signalling transduction pathways. HIFs’ activation is sufficient to change the cell phenotype at multiple levels, by modulating several biological activities from metabolism to the cell cycle and providing the cell with new characteristics that make it more aggressive. In the past few decades, growing numbers of studies have revealed the importance of non-coding RNAs (ncRNAs) as molecular mediators in the establishment of hypoxic response, playing important roles in regulating hypoxic gene expression at the transcriptional, post-transcriptional, translational, and posttranslational levels. Here, we review recent findings on the different roles of hypoxia-induced ncRNAs in cancer focusing on the data that revealed their involvement in tumour growth.

## 1. Introduction

### 1.1. Hypoxia in Solid Tumours

Due to excessive proliferation and abnormal blood vessel formation, most solid tumours are irregularly vascularized and display local regions of hypoxia: reduction of oxygen partial pressure (pO_2_); in these regions will grow clusters of cells with more aggressive phenotypes, high resistance to therapy, and able to affect the surrounding microenvironments [1,2,3]. To survive in hypoxic conditions, tumour cells activate multiple molecular and cellular mechanisms, including alteration in metabolic pathways, alteration in ion channel activity and changes in gene expression through transcriptional and post-transcriptional mechanisms. These biological responses are designed as the hypoxic adaptive responses which activation depends on the function of Hypoxia Hypoxia-Inducible Factors (HIFs).

The transcription factor HIF is a heterodimer consisting of an oxygen-regulated α-subunit (HIF-1α or HIF-2α) and a constitutive β subunit, also known as the aryl hydrocarbon receptor nuclear translocator (ARNT), which presents the bHLH domain required for DNA binding, two PAS domains (PAS-A and PAS-B) essential for dimerization, and one transactivation domain [4]. Among the isoforms, HIF-1α is ubiquitously expressed in various cells and it is considered as the major regulator of oxygen homeostasis, it governs the acute adaptation to hypoxia, whereas HIF-2α expression begins during chronic hypoxia. Both HIF-1α and HIF-2α function as central transcriptional mediators of hypoxic responses, even if with a different pattern [5]; while the role of HIF-3 under hypoxia as well as its mode of action are less clear. Several studies suggested that HIF-3α might be functionally distinct. Specifically, in many studies, it was showed that the HIF-3α, also known as inhibitory PAS domain protein (IPAS), inhibits HIF-1 and HIF-2 expression and/or activity in cell culture [6,7,8] while recent data showed that HIF-3α variants can form αβ dimers that, binding its target genes via the canonical HRE, possess transactivation activity [9].

The reduction of pO_2_ is the key event that switches the hypoxic responses inducing HIF-α activity. At low oxygen levels, HIF-α avoids proteasome degradation and migrates to the nucleus where dimerizes with HIF-1β forming the HIF complex (for a more comprehensive reading on the nuclear transport see [10]). This complex, together with the transcriptional coactivators, will induce genes expression after binding the conserved cis-regulatory site (5′(A/G)CGTG-3′) called Hypoxia Responsive Elements (HREs), on the promoters of hypoxia-regulated genes.

HIF-induced transcriptional regulation changes the cellular phenotype by acting simultaneously on multiple pathways. HIF activity affects cell growth, cell metabolism, redox homeostasis, inflammation, angiogenesis, cell cycle progression, and chemoresistance thus boosting the onset and progression of cancers [11,12,13,14].

Interestingly, in the last years, the literature highlights an increasing number of non-coding RNAs that, induced directly or indirectly by HIF complex, cooperate with it, in establishing the hypoxic responses.

**Figure 1:**
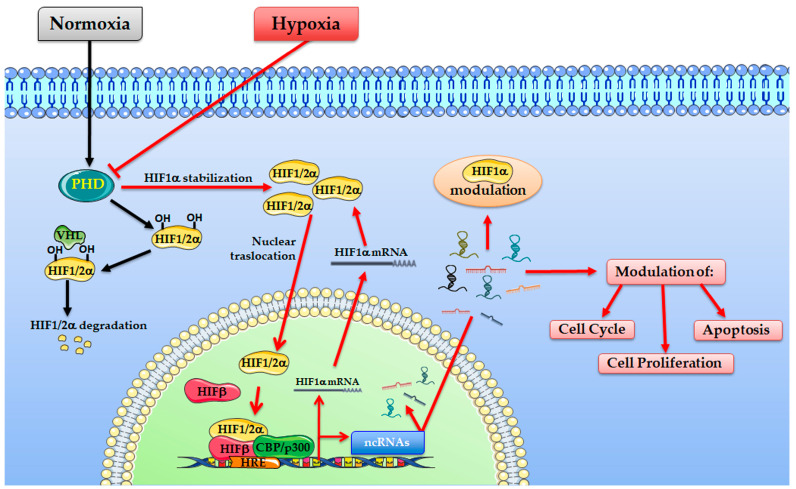
Hypoxia-inducible factor (HIF) complex transcriptionally activates non-coding RNAs (ncRNAs) in response to hypoxia. Under normoxia (black arrows), HIF-1/2α subunits are subjected to hydroxylation by prolyl hydroxylase domain enzymes (PHDs) and other prolyl hydroxylases. Hydroxylated HIF-1/2α subunits are recognized by VHL proteins and targeted for subsequent ubiquitination and proteasomal degradation. Under hypoxia (red arrows), low pO_2_ results in HIF-1/2α accumulation, nuclear translocation and dimerization with HIF-b, finally, after recruitment of CBP/p300, the transcription initiation complex binds the promoter of target genes inducing their expression. Among the hypoxia-induced RNAs, the ncRNAs (miRNAs or lncRNAs) will be involved in different pathways, regulating cell proliferation, cell cycle and cell death. Moreover, some of these can regulate HIF itself.

Here we collected and review the recent discoveries about the role of hypoxia-induced non-coding RNAs in tumour growth, focusing on hypoxia-induced long non-coding RNAs (lncRNAs), and micro RNAs (miRNAs) Figure 1.

### 1.2. Non-Coding RNAs

It is well known that 98% of the human transcriptome encode for different classes of the non-coding RNAs (ncRNAs). According to their size, the ncRNAs are generically classified into small non-coding RNAs (<200 nucleotides, such as miRNA, small nuclear/nucleolar RNA, piwi-interacting RNAs and transfer-RNAs) and lncRNAs (>200 nucleotides). In addition to these two main categories of non-coding RNAs, there is the family of circRNA that differs from the other for functions and structure, characterized by their circular configuration through the 5′ to 3′-phosphodiester bond.

MiRNAs are a class of single-stranded, endogenous small non-coding RNAs containing about 20–25 nucleotides, synthesized from larger RNA transcripts by a complex enzymatic pathway. MiRNAs are transcribed as pri-miRNAs by RNA polymerase II or III, or processed from non-coding RNAs, or from the introns of protein-coding genes (miRtrons) [15]. Pri-miRNAs are then cleaved by the microprocessor complex, formed by the nuclear RNase III enzyme DROSHA and the DGCR8, into a stem-loop structure of 70–110 nucleotides, known as a precursor miRNA (pre-miRNA) [16]. The pre-miRNAs transported to the cytoplasm will be cleaved by the RNase III enzyme/DICER/TRBP2 to generate a 22-nucleotide mature double-stranded miRNA duplex. This miRNA duplex includes the two-strand pair miR-3p/miR-5p, each of which may be selected as functional and recruited by the ARGONAUTE proteins associated with the RNA-induced silencing complex. Single strand miRNAs mainly regulate gene expression at the post-transcriptional level, leading to mRNA degradation or translational repression through partial complementarity to their targets. However, some miRNAs have been found also in the nucleus where they control gene expression at the transcriptional level [17,18,19]. This picture is further complicated by the discovery of RNA modifications that occur in controlling miRNA biogenesis and functions [20].

Long non-coding RNAs (lncRNAs) are a class of RNA transcripts larger than 200 nucleotides in length with no protein-coding capacity [21]. Based on their genomic contexts lncRNAs can be classified into five categories: (I) promoter-associated lncRNAs, (II) enhancer-associated lncRNAs, (III) natural antisense transcripts, (IV) gene body-associated (sense) lncRNAs and (V) long intergenic ncRNAs [22]. LncRNAs genes are very similar to protein-coding genes, presenting marks at their promoters or enhancers, these are transcribed by RNA polymerase II, spliced at canonical splicing sites, and often poly-adenylated [23,24]. Several studies have demonstrated that lncRNAs play critical roles in regulating gene expression at multiple levels, including epigenetic (i.e., the X chromosome silencing, genomic imprinting, and chromatin modification), transcriptional (transcription activation or inactivation), and post-transcriptional level (splicing, mRNA turnover, translation, and RNA interference), through interaction with other biomolecules, such as proteins, regulatory DNA regions, and miRNAs [25,26]. Moreover, the property of each lncRNAs changes according to localization. Nuclear lncRNAs modulate gene expression in cis or in trans, by interacting with transcriptional co-regulators and chromatin remodelling complexes, or impeding transcription factor binding [27]; moreover, by interacting with RNA binding proteins, may control RNA splicing, like for MALAT and NEAT [28]. Into the cytoplasm, lncRNAs bind to various protein partners thus regulating RNA stability, degradation, and translation, moreover they may act as sponges for microRNA thus restoring mRNA targets as demonstrated in several cancer models [29,30].

CircRNAs are mainly formed by “back-splicing” of precursor mRNA (pre-mRNA) and are enriched in the cytoplasm and exosomes [31,32]. These ncRNAs are characterized by a special form with a covalent loop instead of the most common linear which renders them resistant to degradation by RNase R and therefore more stable than linear RNA [33]. According to the source of the genome and biogenesis patterns, circRNAs are divided into four categories: ecircRNAs, ciRNAs, EIciRNA, tricRNAs. The most studied are the circRNAs derived from exons called “ecircRNA”; these are mainly distributed into the cytoplasm, where work as a sponge for miRNAs, allow protein-protein interaction, or in some cases can be translated through a cap-independent mechanism [34,35,36].

Having a look to the other circRNA: EIciRNAs are formed of both exonic and intronic sequences of coding genes [37], “ciRNAs” are circular intronic RNAs, mainly enriched in the nucleus where they are involved in gene regulation [38]. Finally, a special class of intronic circular RNA, “tricRNA” is generated during pre-tRNA splicing [39].

Regardless of their origin, circRNAs have been found to be involved in both physiological and pathological processes through different mechanisms. In the cytoplasm circRNAs are implicated in RNA regulation, or miRNA regulation by acting as competing endogenous RNA [40]; moreover, circRNAs may interact with proteins acting as bridges for protein-protein interaction. In the nucleus, these control gene expressions have been found to be involved in several physiological and pathological process [41,42,43].

## 2. The Hypoxia-Induced Non-Coding RNAs

Hypoxia-responsive ncRNAs have been found to play important roles in hypoxia-driven cancer progression modulating the hypoxic gene expression at transcriptional, and post-transcriptional levels, by acting as effectors of HIF or as direct modulators of the HIF-transcriptional cascade [44,45] (Figure 1).

Profiling techniques and bioinformatics analysis allowed us to unveil more and more hypoxia-regulated non-coding RNA by the presence of the hypoxia response elements (HREs) in their promoter regions [46]. Moreover, several studies have described hypoxic induction of non-coding RNAs lacking HREs indicating an indirect regulation often involving epigenetic mechanisms; HIF may control non-coding RNAs expression through histone deacetylase activation, or affecting miRNA maturation machinery [47,48].

Using microarray analysis on hypoxia-induced gastric cancer cell lines, Wang et al. identified several hypoxia-responsive lncRNAs in gastric cancer. In particular, they found that an intronic antisense lncRNA named lncRNA-AK058003 was among the most induced lncRNAs upon hypoxia treatment in all examined gastric cancer cell lines [49], data confirmed also in breast cancer [50]. In addition, recent data demonstrated that HIF-1α can directly regulate circRNAs at the transcriptional level [51,52] and that HIF-induced circRNAs may promote cancer growth as demonstrated in bladder [53]; however, unlike miRNAs and lncRNAs, the mechanisms of HIF-mediated circRNAs expression have been less investigated and will not be further addressed in this review.

**Figure 2:**
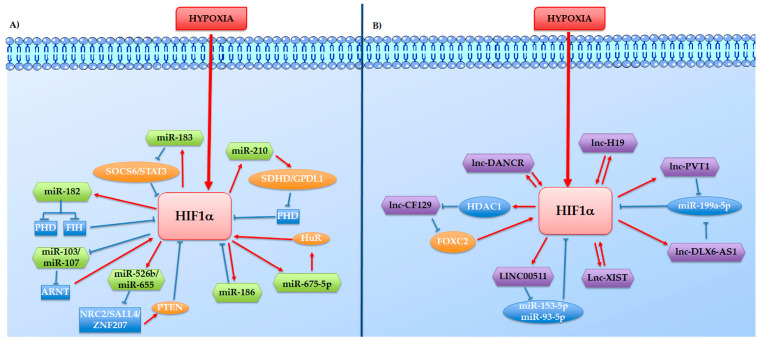
Direct or indirect feedback loops between HIF-1α and hypoxia-regulated ncRNAs. The hypoxia-regulated ncRNAs, HIF-1α, and other co-operators intertwine to form reciprocal feedback loops in both positive and negative manners, represented in the figure respectively with red arrows and blue lines. (**A**) Reciprocal feedback loops between HIF-1α and hypoxia-regulated lncRNAs. (**B**) Reciprocal feedback loops between HIF-1α and hypoxia-regulated miRNAs.

Considering the different mechanisms through which ncRNAs might control tumour growth, these have been divided here into two different groups: (1) the hypoxia-induced ncRNAs that work as HIF effector in promoting cell growth or inhibiting cell death, and (2) the Hypoxia induce ncRNAs such as aHIF-1α, linc-ROR, and lincRNA-p21 which directly or indirectly regulate the HIFs proteins (Figure 2).

### 2.1. The Hypoxia-Induced ncRNAs as HIF’s Effectors in Controlling Cell Viability

#### 2.1.1. Hypoxia-Induced miRNAs with a Role in Tumour Growth

Hypoxic microenvironment can promote tumour growth in a dual mode: by inducing cell cycle deregulation and by allowing apoptosis escape. Several ncRNAs may act as molecular mediators through which, HIF complex controls these processes. Here we collected the most recent and relevant finding of hypoxia-induced miRNAs (hypoxiamiR), further summarized in Table 1.

The most studied hypoxiamiR is the miR-210, it is regulated by HIF in various cell types through the direct binding of the transcription factor to the HREs on its promoter. It was found to represses genes expressed under normoxia, and required to promote tumour growth [84]. The upregulation of miR-210 in solid tumours was associated with bad prognosis, indicating that the target genes affected by miR-210 have a functional impact on tumour malignancy and drug resistance [85,86,87]. Several studies, to date, have demonstrated its involvement in various kinds of tumours affecting a large number of cellular functions, including mitochondrial metabolism, angiogenesis, DNA repair, and cell survival. Wang et al. showed that, in schwannoma cells, hypoxia-induced miR-210 promotes autophagy activation, tumour cell proliferation and angiogenesis, while inhibits apoptosis; intriguingly, in this study, the authors noted that miR-210 promoter region, containing the HREs, was hypermethylated in normoxia, while demethylated in hypoxia thus suggesting a double control on miR-210 expression [54]. Concerning the molecular mediators, firstly, the Grandori group identified in breast and melanoma cancer cells that hypoxia-induced miR-210 targets the Max’s Next Tango (MNT) mRNA, a key transcriptional repressor of the MYC-MAX network. MNT downregulation allows c-MYC to push cells through the cell cycle [56]. Similar results were obtained by Yang and colleagues in glioma stem cells, in which they observed that hypoxia upregulated miR-210 avoided G0/G1 cell cycle arrest via MNT-Max complex-dependent transcription repression [57]. In an in vitro model of ovarian cancer, Li et al. demonstrated that hypoxia upregulates miR-210 thus promoting tumour cell proliferation and cell clone generation via targeting PTPN1 (tyrosine-protein phosphatase non-receptor type 1) and inhibiting apoptosis [58]. It is notable that several studies highlighted the predicted miR-210 seed sites in apoptosis-related mRNA transcripts such as AIFM3 (Apoptosis-Inducing Factor Mitochondrion-associated 3), CASP8AP2 (Caspase-8-Associated Protein-2) and SIN3A (a transcription repressor that forms a complex with histone deacetylase 1) [59,60,88,89].

Recently, an interesting manuscript of Du et al. demonstrated that hypoxia-induced miR-210-3p controls cell proliferation by promoting Warburg effects and, concomitantly by inhibiting p53 activity in triple-negative breast cancer [61].

Taken together, these studies support a model in which, through the regulation of a single miRNA, HIF can simultaneously target multiple factors with a key role in the apoptotic process, ultimately promoting uncontrolled cell proliferation and carcinogenesis.

Another highly expressed hypoxiamir, regulated by both HIF-1 α and HIF-2α, is miR-21. It is considered to act as oncomirs by targeting many tumour suppressor genes involved in cell proliferation, apoptosis, and invasion in several types of cancer [69,70]. The pro-oncogenic role of miR-21 was in deep studied in the last years since to propose anti-miR-21 as a strategy to fight tumour growth [90]; here is interesting to note that hypoxia-induced miR-21 shows an elevated level in hypoxic exosomes. Li et al., demonstrated that tumour-derived exosomes enriched in miR-21 are internalized by normoxic cells driving recipient cells toward a pro-metastatic phenotype [91].

One of the miRNAs, which expression increases in the hypoxic tumour, is the miR-675. It was found up-regulated by HIF in several tumours including Glioblastoma [75,76] and colorectal cancer [77]. Recently it was described its role in controlling cell cycle by regulating Glycogen Synthase Kinase 3β (GSK-3β) activity and allowing β-catenin nuclear localization [78]. MiR-675 was found up-regulated in hypoxic non-small cell lung cancer where in addition to promoting cell proliferation, it acts on apoptosis by directly inhibiting the expression of p53 [79]; also in this case, through the induction of a single miRNA, HIF promotes tumour growth by acting simultaneously on several molecular pathways.

In a study conducted by Zhao et al., miR-191 was found to be upregulated by HIF-1α. They showed that miR-191 promoted the proliferation and migration of non-small cell lung cancer cells (NSCLC) by targeting of NF1A (Nuclear factor 1 A-type) under chronic hypoxic conditions [64]. In breast cancer, Nagpal and colleagues showed that miR-191 is upregulated by both HIF-1α and HIF-2α and its overexpression is responsible for cancer aggressiveness by promoting cell proliferation, and survival under hypoxia; moreover, they demonstrated that miR-191 promotes TGFβ expression thus revealing a molecular link between HIF and TGFβ signalling pathways, both pivotal in the regulation of breast cancer metastasis [63].

He et al. highlighted the role of HIF-1α in transcriptional upregulation of the miR-224 in gastric cancer cells. Through target gene validation, these authors revealed that miR-224 directly targets RASFF8 (Ras association domain family member 8), stimulating p65 nuclear translocation and NF-kB transcriptional activity to confer gastric cancer cells with more aggressive phenotype [71]. Their results suggest that hypoxia-inducible miR-224 promotes gastric cancer cell growth by downregulating RASSF8 and acts as an oncogene, implying that inhibition of miR-224 may have potential as a therapeutic target for patients with hypoxic gastric tumours [71].

Several studies conducted on gastric cancer cells using different approaches (i.e., ChIP assay, luciferase assay, as well as qRT-PCR) revealed the direct relationship between HIF-1α and different miRNAs. Ge et al. observed that miR-421, up-regulated by HIF-1α, can promote tumour behaviour in gastric cancer by targeting the caspase-3 thus inhibiting apoptosis [80]. Zhao and colleagues revealed that HIF-1α can directly bind the miR-27a promoter increasing the activity of some antiapoptotic pathways (i.e., the MDR1/P-gp, Bcl-2, LRP) and promoting multidrug resistance [65].

It is notable that while several miRNAs are directly induced by HIF, a great number of non-coding RNAs were found to be down-regulated in hypoxic conditions. 

Liu et al. showed that in hepatocellular carcinoma miR-204 expression is inhibited by HIF-1α. These authors proved that hypoxia-induced down-regulation of miR-204 promotes malignant transformation through the up-regulation of Vasodilator-stimulated phosphoprotein (VASP); this is a regulator of cytoskeletal actin and cell migration, and its expression correlates with aggressive phenotype and metastasis both in vitro and in vivo [81]. MiR-33a and miR-199a-5p are found to be reduced under the regulation of HIF-1α in HCC [82,83]. Li et al. proved that mirR-199a-5p inhibition by HIF-1α regulates Warburg effect and induce tumour cell proliferation in hepatocellular carcinoma [83]. Also, miR-548an, a tumour suppressor miRNA, is down-regulated by HIF-1α in pancreatic cancer cells, and it is involved in increasing vimentin level and facilitating the pancreatic tumorigenesis [73].

Intriguingly, several studies have reported hypoxamirs working as tumour suppressors e.g., the miR-145 or the miR-215 [62,92], thus suggesting the existence of an intricate network of interactors not yet fully revealed. To solve this, some aspects that have been neglected so far must be taken into account: (i) here we reviewed only a fraction of HIF-induced miRNA but several other miRNAs are induced under hypoxic condition in a HIF-independent manner, (ii) a single miRNA has numerous targets in the same cell, and (iii) hypoxia-induced lncRNAs can sequester multiple miRNAs preventing them from reaching their targets.

#### 2.1.2. Hypoxia-Induced lncRNAs Participating in Tumour Growth

The first reported hypoxia-inducible noncoding RNA is the transcript generated by the maternally imprinted oncofetal gene *H19*, for the full list see Table 2.

It is well known that lncRNA H19 is expressed aberrantly in a wide range of tumours, promoting growth and dissemination (see review [117]); moreover, in different tumours, e.g., prostate cancer and glioblastoma, H19 expression was found significantly upregulated in hypoxia [75,93,94,118]. Firstly, Matouk et al. showed that H19 expression is dependent on the mutational status of the tumour suppressor p53. Specifically, during hypoxia, H19 expression is not induced in p53 wild type cells, while it is significantly increased in p53 null cells, indicating that lncRNA expression under hypoxia could be influenced by p53 mutation status [95]. Recently the crosstalk between lncH19 and p53 was further investigated in lung cancer, where it was demonstrated that lncH19, through upregulation of its intragenic miR-675, controls p53 expression [79,96].

Although several elements might contribute to controling lncH19 gene expression, it has been demonstrated that HIF-1 directly induces H19 expression [94].

The studies conducted by Wu and colleagues confirmed that HIF-1α is actively involved in lncRNA H19 modulation in vitro and in vivo, proving that PTEN down-regulation favours hypoxia-driven H19 induction. Moreover, they found that HIF-1α may promote H19 expression by both directly binding to the H19 promoter and indirectly through SP1-mediated H19 transcriptional activation under hypoxia in glioblastoma cells [94].

Another of the lncRNAs induced by hypoxia is HOTAIR (HOX transcript antisense intergenic RNA). HOTAIR is an oncogenic lncRNA and a negative prognostic factor for several types of cancer, such as non-small cell lung cancer (NSCLC), breast, cervical, and endometrial cancer [97,98]. Zhou et al. reported that, in NSCLC cells, the upregulation of HOTAIR under hypoxia is directly dependent on HIF-1α, and that HIF-induced HOTAIR increases cell viability and invasion, while inhibits apoptosis of hypoxic cells [99]. Moreover, it was previously showed that HOTAIR can downregulate the expression of different tumour suppressor genes including JAM2, PCDH10, and PCDHB5 [100].

LncRNA-UCA1 (long non-coding transcript named urothelial carcinoma associated 1), in hypoxic urinary bladder cancer, is regulated in both HIF dependent and HIF independent manner with a role in hypoxia-mediated cancer cell proliferation and invasiveness. In particular, it was observed that LncRNA-UCA1 knockdown in hypoxia can reduce tumours’ invasiveness by modulating apoptosis-associated proteins; LncRNA-UCA1 inhibition downregulates the anti-apoptotic protein Bcl-2 and upregulates the pro-apoptotic protein Bax [101]. Oncogenic functions of lncRNA-UCA1 have been reported in several cancer types, including colon cancer, breast cancer, and melanoma moreover, it is interesting to note that, as well demonstrated by Xue et al., this lnc can be transported by exosomes released by hypoxic bladder cancer, prompting tumour growth once internalized by normoxic cells [119].

The different data recently collected on the lncRNA MALAT1 (metastasis-associated lung adenocarcinoma transcript 1) are an example of how a single lncRNA can simultaneously activate several strategies that promote the same phenomenon, in this case, tumour progression.

Choudry et al. have first identified lncRNA MALAT1 as HIF-induced lncRNA in MCF-7 breast cancer cells [47], while its mechanisms of action were identified more recently. In hepatocellular carcinoma, Zhou et al. demonstrated that hypoxia-induced MALAT1 participates in cells proliferation, apoptosis, migration, and invasion by sponging miR-200 [102]. It is well known in fact that upregulation of miR-200a reduces cell proliferation, migration, and invasion, while increases apoptosis. Interestingly, in lung adenocarcinoma cells A549, lncRNA MALAT-1 is significantly increased after hypoxic stimulation and promotes proliferation, migration and invasion cells through a different molecular mechanism [103]. The authors showed that upregulated lncRNA MALAT-1 interacts with PTB-associated splicing factor protein (PSF), this is responsible for epigenetic regulations of its target genes, such as the proto-oncogene G antigen 6 (GAGE6). The binding of MALAT-1 releases PSF from its downstream proto-oncogene and activates its transcription thus finally promoting proliferation, migration and invasion in A549 cells [106].

Moreover, once induced by hypoxia, MALAT1 regulates itself in myeloma. Ikeda and colleagues demonstrated that it could maintain and promote its expression under a hypoxic microenvironment through the regulation of the KDM3A (H3K9 demethylating enzyme) that in turn promotes MALAT1 transcription [104].

The lncRNA NEAT1, as reported by Choudhry et al., was significantly induced during hypoxia in MCF-7 cells by HIF direct binding on NEAT1 promoter [47]. NEAT1 is a pivotal architectural component of paraspeckles, which are involved in regulating gene/protein expression, either by protein sequestration or RNAs editing with subsequent retention in the nucleus. During hypoxia, this lncRNAs plays a role in controlling mRNAs involved in increased tumorigenesis, cell proliferation, cell survival and apoptosis inhibition [120]. Kong and colleagues identified miR-101-3p as a direct target of NEAT1. In particular, these authors observed that NEAT1 by sponging miR-101-3p can induce SOX9 upregulation that is involved in enhanced Wnt/β-catenin signalling. In this study, the authors supposed that NEAT1 has an oncogenic role in NSCLC progression via miR-101-3p/SOX9/Wnt/β-catenin axis [105].

Recently, Huan et al. identified the long non-coding LUCAT1 as a new player for hypoxic responses in CRC cells [106]. Under hypoxia, LUCAT1 is transcribed by HIF-1α and it is involved in regulating cell growth, apoptosis, and DNA damage in CRC cells. Moreover, LUCAT1 overexpression induces chemoresistance in CRC cells both in vitro and in vivo. In particular, they observed that hypoxia-induced LUCAT1 is located in the nucleus of CRC cells, where it physically interacts with the RNA-binding protein PTBP1 modulating mRNA alternative splicing pathway of several PTB1-downstream transcripts including CD44. It is known, in many cancers, that alternative spliced CD44 (CD44v, retained form) can induce apoptosis resistance, prolonging G2/M phase, and DNA damage resistance. Thus, the authors identified the LUCAT1′s role in alternative splicing control in cancer [106].

Another hypoxia-regulated lncRNA controlling cell proliferation is the LncRNA AGAP2-AS1, an antisense lncRNA located at 12q14.1 and dysregulated in cancers [121]. LncRNA AGAP2-AS1 promotes cell proliferation and inhibits apoptosis in gastric cancer and HCC [107,122]. It was demonstrated that AGAP2-AS1 sponges miR-16-5p that exerted a suppressive effect on cell proliferation, migration, invasion, and EMT progress of HCC cells. In addition, miR-16-5p can target Annexin A11 that, mediating the downstream phosphorylation of AKT, is associated with cancer progression, metastasis, apoptosis, and cell growth [107].

LncRNA PVT1 was recently found to induce cell proliferation through cell cycle modulation in many cancers [123,124]. Wang and colleagues confirmed that PVT1 was overexpressed in the hypoxic lung cancer cells and it was involved in cell growth and proliferation, demonstrating that PVT1 knockdown could significantly suppress lung cancer cell proliferation in vitro [108]. In another study, the results obtained by Wang and colleagues revealed that this lncRNA may act as an oncogene in nasopharyngeal carcinoma tumorigenesis [109].

Recently, another hypoxia-responsive lncRNA HIF1A-AS2 was revealed to be involved in a regulatory mechanism in tumorigenesis of breast cancer [110]. Guo and colleagues showed that HIF1A-AS2 was upregulated in breast cancer cells and tumour biopsies. Moreover, the reduction of HIF1A-AS2 significantly inhibited the cell proliferation, invasion, and angiogenesis both in vitro and in vivo. Furthermore, the results of their experiments in xenograft nude mice indicated that silencing of HIF1A-AS2 inhibited tumour growth and motility by targeting miR-548c-3p through regulating HIF-1α/ VEGF signalling pathway in vivo [110].

It is notable that some lncRNAs have been found down-regulated by HIF, an example is the lncRNA-LET [111], which usually functions as a tumour-suppressing element. Ma et al. reported that lncRNA-LET was significantly downregulated in gallbladder cancer cell lines under hypoxia, and its downregulation can induce cell viability and proliferation under hypoxic conditions. Furthermore, cell cycle analysis indicated that lncRNA-LET could inhibit gallbladder cancer cell proliferation by upregulating G0/G1 arrest and apoptosis [111]. Much of the data concerning the role of lncRNAs in driving the hypoxic response is centred on lncRNA-miRNAs interaction. It would be interesting, in this case, to analyse how the expression panel of cellular miRNAs varies in response to hypoxia-induced down-regulation of lncRNA–LET, this could lead to new mechanisms for the inhibition of the hypoxia-induced miRNAs.

## 3. Feedback Loops between HIF and the Hypoxia-Induced Non-Coding RNA

Nowadays, several studies reported that the reciprocal interplay that occurs between miRNAs and HIF transcription complex has a fundamental role in tumorigenesis. Bioinformatic analyses revealed that several miRNAs can target the 3′UTR of HIFs’ RNAs modulating their expression. Moreover, several studies have demonstrated that miRNAs could control HIF through an indirect mechanism. Hypoxia-induced miRNAs have been shown to target genes that may affect HIF-1α stability, thus forming a positive feedback loop. So far, these miRNAs include the miR-210 miR-199a, miR-18a, miR-183 and miR-138.

As widely described in this review miR-210 is a significantly upregulated miRNA in almost all cancer cell lines under hypoxic condition. Increased miR-210, other than mediating hypoxic responses, can stabilize HIF expression. It was demonstrated that miR-210 targets the electron transport chain through SDHD (succinate dehydrogenase complex, subunit D), inducing accumulation of succinate that, in turn, inhibits Prolyl hydroxylase domain enzymes (PHD) and stabilizes HIF-1α, thus forming a positive-autoregulatory loop [125]. More recently, Du et al. demonstrated that miR-210-3p targets GPD1L (glycerol-3-phosphate dehydrogenase 1-like) and consequently affects HIF1α stability inducing an effect similar to that observed with SDHD inhibition [61].

Data collected from prostate cancer demonstrated that hypoxia-induced overexpression of miR-182 can result in reduced levels of two negative regulators of HIF1 signalling: the PHD and the Factor inhibiting HIF-1 (FIH1) [126].

Also, the miR-675-5p, which is embedded in hypoxia-induced long non-coding RNA H19, is required to sustain the activity of HIF-1α at least in Glioblastoma and Colorectal cancer models [75,77]. It was demonstrated that miR675-5p is a hypoxia-regulated miRNA essential for hypoxia establishment and involved in hypoxia-mediated angiogenesis. Specifically, we demonstrated that miR675-5p inhibition during hypoxia affects HIF-1α activity. In particular, the proposed model indicated that miR675-5p sustains HIF-1α and VEGF mRNA stabilization trough the involvement of HuR, an RNA binding protein that binds HIF-1α and VEGF mRNAs, increasing their stability.

Another two positive feedback loops have been found to coexist in multidrug-resistant hepatocellular cancer cells, HIF-1α/miR-183/IDH2/HIF-1α and HIF-1α/miR-183/SOCS6/p-STAT3/HIF-1α, which may modulate HIF-1α protein [127].

Recently, in silico bioinformatics analysis showed that hypoxia-upregulates miR526b/miR655 stabilize HIF proteins targeting the transcription factors NR2C2, SALL4, and ZNF207 that, in turn, regulate PTEN (a negative regulator of HIF-1α) and NFκB1 (positive regulator of COX-2 and EP4) [128].

An interesting loop is that mediated by the down-regulation of two miRNAs: miR-103 and miR-107 [129,130], that do not target HIF-1α but ARNT, the binding partner of the HIF complex. Although widely considered to be constitutively expressed, ARNT was found to be regulated by hypoxia in several models where it confers drug-resistance and tumour growth [131,132,133]. Moreover, Mandl et al. demonstrated that ARNT over-expression, alone, can stabilize HIF-1a and activate HRE-equipped genes in normoxia, providing evidence that little is known yet about the role of ARNTs in regulating the hypoxic response [131].

In the last years, to identify new strategies to block hypoxic responses, many miRNAs have been tested for targeting HIF activity. Indeed, according to a recent study, in CRC cell lines it was suggested that the ectopic expression of miR-1 has an anti-proliferative effect by targeting HIF-1α [134]. In pancreatic cancer cells, over-expression of miR-142, normally reduced under hypoxic condition, significantly affects cell proliferation and migration by downregulating HIF-1α [135]. In another study, Liu et al. showed that miR-186/ HIF-1α axis is fundamental to the proliferation of gastric cancer cells, and that upregulation of miR-186 can inhibit tumour cell proliferation through targeting HIF-1α [136].

Also, several lncRNAs have been found involved in the positive loops stabilizing HIF. Among this, it is possible to identify two major groups: (i) the lncRNAs that sponge miRNAs targeting HIFs and (ii) the lncRNAs that directly participate in HIF genes transcription or mRNA stabilization. However, it is notable that some lncs have been identified to work through both mechanisms, for example, the plasmacytoma variant translocation 1 (PVT1).

Firstly, Wang and collaborators revealed a connection among lnc-PVT1, miR-199a-5p, and HIF-1α in cell response to hypoxia; they demonstrated that hypoxia-induced PVT1 could function as a molecular sponge inhibiting miR-199a-5p and thus promoting the expression of its endogenous target HIF-1α [108]. Further studies conducted on nasopharyngeal carcinoma, proposed a new putative mechanism by which PVT1 could stabilize HIF-1α [109]; here, the authors demonstrated that the lncRNA functions as a scaffold for the chromatin modification factor KAT2A, which stabilizes HIF-1α via H3K9ac/TIF1β complex-mediated NF90 (a double-stranded RNA-binding protein) transcriptional activation [109].

Several lncRNAs work as competitive endogenous RNAs, modulating the amount of miRNA available to interact with HIF’s messenger. In colorectal cancer, the hypoxia-induced lncRNAs XIST and LINC00511 down-regulate respectively the miR-93-5p and the miR-153-5p, which both have HIF-1α mRNA among their targets [137,138]. In nasopharyngeal carcinoma, the hypoxia-indued lncRNA DLX6-AS1 (long-chain non-coding growth stasis specific protein 6 antisense RNA1) stabilizes HIF-1α mRNA sponging the miR-199a-5p [139]. A further positive molecular loop is that mediated by the expression of the hypoxia-induced H19 that stabilizes HIF-1α mRNA competing with the miR-138 [140].

Concerning the lncRNAs that directly modulate HIF1 expression, in nasopharyngeal carcinoma HIF-1α mRNA stability is mediated by the lncRNA DANCR (differentiation antagonizing non-protein coding RNA); this is required for the interaction between HIF-1α mRNA and the complex NF90/NF45 which was found increase HIF-1α mRNA stability [141]

Moreover, some lncRNAs, down-regulated during hypoxic conditions, have been found able to destabilize HIFs.

In pancreatic cancer cells, Liu et al. showed that HIF transcriptionally downregulates lnc-RNA-CF129 expression by recruiting HDAC1 to its the promoter [142]. Interestingly, the authors demonstrated that CF129 can down-regulates the transcription factors FOXC2, that promotes HIF-1α expression [142].

Another negative regulator of HIF-1α is the lncRNA HITT (HIF-1α inhibitor at translation levels) that plays roles in modulating hypoxia-mediated angiogenesis and tumour growth in vivo. Recently, Hu and collaborators demonstrated that HITT inhibits HIF-1α transcription by guiding Ezh2 through the formation of an RNA-DNA triplex with the HIF-1α promoter. The occupancy of Ezh2 and its substrate H3K27me3 on the HIF-1α promoter is detected under normoxia and is reduced by hypoxia [143].

Park and his collaborator identified a new way by which the prolyl isomerase Pin1 plays a role in the regulation of HIF-1α. Interestingly they found that the PNI1 transcript variant 2 inhibits HIF-1α transcription hiding the binding site for NFAT on the HIF1A promoter [144].

## 4. Conclusions

In conclusion, we reviewed here the most recent findings of hypoxia-induced non-coding RNA supporting HIF in the cell growth control. The data collected revealed a complex network of molecular interactors activated directly by the HIF complex in oxygen deficiency. The picture becomes even more complicated if we add to these the HIF-independent molecular pathways, which also take part in the hypoxic response. More detailed knowledge of these pathways and their interactions is necessary to identify new and more effective therapeutic targets.

## Figures and Tables

**Table 1:** List of hypoxia-responsive miRNAs involved in cell proliferation, apoptosis and cell cycle regulation.

miRNAs	Cancer Types	Regulation Hypoxia-Mediated	Targets	Functions	References
miR-210	Schwannoma cells	upregulation	NA	enhances tumour cell proliferation	[54]
	neuroblastoma cells	upregulation	Bcl-2	induces apoptosis	[55]
	Breast and melanoma cancer cells,	upregulation	Max’s Next Tango (MNT)	inhibits hypoxia-induced cell cycle arrest	[56]
	Glioma stem cells	upregulation	MNT-Max complex	inhibits hypoxia-induced cell cycle arrest	[57]
	Epithelial ovarian cancer	upregulation	PTPN1	promotes cell proliferation and inhibits apoptosis	[58]
	Epatoma cells	upregulation	AIFM3	inhibits hypoxia-induced cell cycle arrest	[59]
	Glioma cells	upregulation	SIN3A	inhibits proliferation and promotes apoptosis	[60]
	triple-negative breast cancer	upregulation	p53	promotes cell proliferation	[61]
miR-210-3p	bladder cancer	upregulation	NA	induces apoptosis	[62]
miR-145	breast cancer	upregulation	TGFb2, HuR	promotes proliferation	[63]
miR-191	non-small cell lung cancer cells	upregulation	NF1A	promotes proliferation	[64]
	gastric cancer	upregulation	MDR1/P-gp, LRP and Bcl-2 pathways	promotes proliferation	[65]
miR-27a	gastric cancer	upregulation	PTEN	promotes proliferation	[66]
miR-382	breast cancer	upregulation	PDCD4	inhibits apoptosis	[67]
miR-424	colorectal cancer	upregulation	DAPK, KLF4	promotes hyperproliferation and decreases apoptosis	[68]
miR-103/107	pancreatic cancer cells	upregulation	NA	promotes proliferation and inhibits apoptosis	[69]
miR-21	cervical cancer cells	upregulation	PTEN/AKT pathway	promotes cell growth	[70]
	gastric cancer	upregulation	RASSF8	promotes cell growth	[71]
miR-224	bladder cancer cells	downregulation	FGFR3	upregulates proliferation	[72]
miR-100	pancreatic cancer cells	downregulation	Vimentin	inhibits proliferation	[73]
miR-548an	acute myeloid leukaemia cells	downregulation	p21, STAT3	inhibits cell growth	[74]
miR-101	glioblastoma	upregulation	NA	promotes cell proliferation	[75,76]
miR-675	colorectal cancer cells	upregulation	β-catenin localization	regulates cell cycle	[77,78]
	non-small cell lung cancer	upregulation	p53	promotes cell proliferation	[79]
	gastric cancer	upregulation	Caspase 3	inhibits apoptosis	[80]
miR-421	hepatocellular carcinoma	downregulation	VASP	promotes cell growth	[81]
miR-204	hepatocellular carcinoma	downregulation	TWIST1	induces tumour cell proliferation	[82,83]
miR-33a	hepatocellular carcinoma	downregulation	NA	upregulates tumour cell proliferation	[82,83]

**Table 2:** List of hypoxia-responsive long non-coding RNAs involved in cell proliferation, apoptosis and cell cycle regulation.

lncRNAs	Cancer Types	Regulation Hypoxia-Mediated	Regulatory Mechanism	Targets	Functions	References
H19	colorectal cancer, glioblastoma, non-small cell lung cancer and lung cancer	upregulation	direct transcriptional activation or indirect SP1-mediated transcriptional activation	NA	mediates cancer cell proliferation upregulates cell viability	[79,93,94,95,96]
HOTAIR	NSCLC, breast, cervical and endometrial cancer	upregulation	transcriptional activation	JAM2, PCDH10, PCDHB5	upregulates cell viability	[97,98,99,100]
UCA1	urinary bladder cancer	upregulation	transcriptional activation	BAX	mediates cancer cell proliferation	[101]
MALAT1	hepatocellular carcinoma	upregulation	transcriptional activation	miR-200a	upregulates cell proliferation	[102]
	lung adenocarcinoma cells	upregulation	transcriptional activation	PTB-associated splicing factor protein (PSF)	promotes proliferation	[103]
	myeloma cells	upregulation	transcriptional activation	MALAT1 itself through the regulation of the KDM3A (H3K9 demethylating enzyme)	upregulates cell proliferation	[104]
NEAT1	NSCLC	upregulation	transcriptional activation	miR-101-3p	increases cell proliferation	[105]
LUCAT1	colorectal cancer	upregulation	transcriptional activation	PTBP1	induces apoptosis resistance, prolonging G2/M phase	[106]
AGAP2-AS1	gastric cancer and HCC	upregulation	transcriptional activation	miR-16-5p	promotes cell proliferation and inhibits apoptosis	[107]
PVT1	lung cancer cells, nasopharyngeal carcinoma	upregulation	NA	NA	induces cell proliferation	[108,109]
HIF1A-AS2	breast cancer cells and tumour	upregulation	transcriptional activation	miR-548c-3p	induces cell proliferation	[110]
LET	gallbladder cancer cells	downregulation	NA	NA	induces cell viability and proliferation	[111]
Hincut 1	colorectal/breast cancer cells	upregulation	transcriptional activation	NA	upregulates proliferation	[112]
Linc-ROR	liver cancer cells	upregulation		miR-145	promotes cell survival	[113]
BX111	pancreatic cancer	upregulation	transcriptional activation	ZEB1	promotes cell proliferation	[114]
FALEC	prostate cancer	upregulation	transcriptional activation		promotes cell proliferation	[115]
NUTF2P3-001	pancreatic cancer	upregulation	transcriptional activation	NA	promotes cell viability and proliferation	[116]
